# The impact of the 2021 Thrifty Food Plan benefit re-evaluation on SNAP participants’ short-term food security and health outcomes

**DOI:** 10.3389/fpubh.2023.1142577

**Published:** 2023-06-29

**Authors:** Cindy W. Leung, Julia A. Wolfson

**Affiliations:** ^1^Department of Nutrition, Harvard T. H. Chan School of Public Health, Boston, MA, United States; ^2^Department of International Health, Johns Hopkins Bloomberg School of Public Health, Baltimore, MD, United States; ^3^Department of Health Policy and Management, Johns Hopkins Bloomberg School of Public Health, Baltimore, MD, United States

**Keywords:** supplemental nutrition assistance program, food stamps, food insecurity, diet quality, mental health

## Abstract

**Introduction:**

The Supplemental Nutrition Assistance Program (SNAP) is the largest federal nutrition assistance program in the United States, and played a critical role in mitigating food insecurity during the COVID-19 pandemic. In 2021, the updated Thrifty Food Plan (TFP), which provides the basis of SNAP benefit allotments, led to a 21% monthly benefit increase for SNAP participants. The objective of this study was to examine the effects of the TFP re-evaluation on food insecurity, diet quality, and mental health using a natural experiment design.

**Methods:**

A longitudinal, web-based study was conducted among 1,004 United States adults with incomes at or below $65,000 in September 2021 (prior to the policy change) and February 2022 (after the policy change). Outcomes of interest included household food security, diet quality, perceived stress, and anxiety/depression, assessed using validated instruments. We used difference-in-differences regression modeling to assess the effects of the policy change on participants’ outcomes, adjusting for sociodemographic covariates. Qualitative responses to open-ended questions about the policy change were analyzed using thematic analysis.

**Results:**

Prior to the policy change, SNAP participants had significantly worse food insecurity, lower diet quality scores, and higher perceived stress and anxiety/depression when compared to non-participants (all *P*s < 0.05). After adjustment for differences in sociodemographic characteristics, there were no significant effects of the TFP re-evaluation on food insecurity, diet quality, and mental health outcomes among SNAP participants relative to non-participants (all *P*s > 0.05). Qualitative responses suggested that rising food prices and growing inflation potentially negated the benefits of the policy change; however, most SNAP participants described the added benefits as helpful in purchasing additional food supplies and offsetting other household costs during this period.

**Discussion:**

The TFP benefit increase may have helped to prevent inflation-related disparities in food insecurity and health outcomes from widening among SNAP participants and non-participants. Further research is needed to determine the long-term impacts of this policy change.

## Introduction

The COVID-19 pandemic has exerted an intense and far-reaching pressure on economies, food systems, and supply chains around the world. Food insecurity is one important area that continues to be impacted. In the United States (US), studies conducted in the early months of the pandemic showed significantly elevated levels of food insecurity particularly among Black and Hispanic adults, in families with children, and those who were newly unemployed (1–4). In 2021, 10.2% of United States households–or nearly 34 million Americans–experienced food insecurity (5). Food insecurity is an important social determinant of health with long-lasting consequences. Research shows that food insecurity has significant adverse effects on chronic physical and mental health outcomes among low-income adults (6, 7). Thus, over the course of the pandemic, national food, nutrition, and economic policies have been swiftly enacted to mitigate drastic increases in food insecurity and prevent associated health consequences.

The Supplemental Nutrition Assistance Program (SNAP) has played a critical role in alleviating food insecurity in the United States since the onset of the pandemic (8). SNAP is the largest federal food assistance program administered by the United States Department of Agriculture (USDA) and operates by providing low-income families with monthly benefits to purchase food (9). The benefits are loaded onto an Electronic Benefits Transfer (EBT) card and can be redeemed at over 200,000 grocery stores and retailers nationwide. Prior to the pandemic, SNAP has been shown to improve food insecurity, reduce poverty, and stimulate economic growth (10–12). Thus, in order to alleviate widespread food insecurity, multiple policies were passed to temporarily increase and expand SNAP benefits over the course of the pandemic. Specifically, from March 18, 2020 through the end of the state or national public health emergency, USDA allowed states to provide emergency allotments to increase participants’ benefit levels to the maximum amount for their household size ($680/month for a household of four) (13). From January 1, 2021 to September 30, 2021, the American Rescue Plan of 2021 raised benefit amounts by an additional 15% (e.g., mean $27/day per person increase) (14). While these policies were motivated by the pandemic-related public health emergency, another permanent action via White House Executive Order was a re-evaluation of the Thrifty Food Plan (TFP), the low-cost food plan on which SNAP benefit allotments are based (15). The USDA defines the TFP as “the cost of groceries needed to provide a healthy, budget-conscious diet for a family of four” (16). As part of the 2018 U.S. Farm Bill, Congress ordered the United States Department of Agriculture to reexamine the TFP to bring it up to date with current food prices, national dietary guidance, nutritional content, and typical dietary patterns of low-income families. As a result, in August 2021, the USDA announced a modernization of the TFP, which had not been updated since 2006 (17). Due to the TFP re-evaluation, SNAP benefits would be increased by 21% per month (approximately $145 for a family of four), effective October 1, 2021 (18).

This study used the national TFP re-evaluation as a natural experiment to understand the initial effects of this policy change on SNAP participants’ health and well-being. Specifically, the objective of this study was to examine the impacts of the increase in SNAP benefit levels due to the TFP re-evaluation on food insecurity, diet quality, and mental health among SNAP participants compared to low-income non-participants over the same time period.

## Methods

### Study design and participants

We designed a longitudinal, web-based (Qualtrics) study to evaluate the TFP re-evaluation on SNAP participants’ outcomes. Participants were recruited through CloudResearch, formerly TurkPrime, an online crowdsourcing platform that works through Mechanical Turk (MTurk) (19). MTurk is microtask platform that connects researchers to “workers” or individuals interested in completing surveys or performing other tasks. CloudResearch assists researchers in recruiting populations from hard-to-reach populations (e.g., SNAP participants) and has been widely used for academic research since 2010 (20–26). In this study, we recruited a sample of US adults with incomes ≤$65,000 to complete two surveys, the first in September 2021 (i.e., the month before the initiation of the TFP increase) and second in February 2022 (i.e., 4 months after the initiation of the TFP increase). In total, 1,776 respondents completed the baseline September 2021 survey. All respondents were then invited back in February 2022 to complete the endline survey. At follow-up, 1,195 adults responded, yielding a 67% retention rate. To avoid deterring or attracting participants based on their SNAP participation status, the purpose of the survey was described as “[understanding] the effect of the COVID-19 outbreak on the health and wellbeing of adults in the United States.” Survey respondents were compensated $3 for the baseline survey and $6 for the endline survey by CloudResearch.

### Supplemental Nutrition Assistance Program participation

Survey respondents were asked: “Are you currently receiving benefits from any of the following government programs?” Response options included WIC (Women, Infants, and Children), SNAP or the Food Stamp Program, TANF (Temporary Assistance for Needy Families), or SSI (Supplemental security income). Respondents who checked “SNAP” in both surveys were categorized as current SNAP participants (*n* = 415). Respondents who did not check “SNAP” in either survey were categorized as non-participants (*n* = 589).

Adults whose SNAP participation status changed between the baseline and endline surveys were excluded from the analyses to avoid contamination of SNAP participation groups: 34 adults were removed because they initiated SNAP participation between surveys and 145 adults were removed because they ceased SNAP participation between surveys. An additional 12 adults were removed because of missing data on the endline SNAP question.

### Outcomes

Household food security was measured over the past 30 days using the 18-item U.S. Department of Agriculture Household Food Security Survey Module (HFSSM) (27). This scale is widely used for food insecurity surveillance and research, and considered the “gold standard” measure of household food security status in the U.S. Briefly, questions are ordered by severity and assess experiences and behaviors related to food-related hardship. Ten questions pertain to experiences of adults and eight questions pertain to experiences of children (which are omitted if no children reside in the household). Affirmative responses are summed to create a total food security score. Per USDA guidelines, food insecurity is defined as a score of three or higher. As a sensitivity analysis, we also considered the continuous HFSSM score.

Dietary intake was assessed over the past 30 days using the Prime Diet Quality Score (PDQS-30D) (28). The PDQS is a validated food-based diet quality index developed to identify dietary patterns that reduce the risk of major chronic diseases. The PDQS assesses the intake of 22 foods/food groups (14 healthy, seven unhealthy, and one neutral), across seven frequency categories (<1 time/month, 2–3 times/month, 1–2 times/week, 3–4 times/week, 5–6 times/week, 1 time/day, ≥2 times/day). Responses are coded from zero to six, with unhealthy items reverse-scored and the neutral item not scored. A summary score is created ranging from 0 to 126, with a higher score denoting an overall healthier dietary pattern. We further categorized the overall PDQS score into “healthy” components (fruits, vegetables, whole grains, nuts, legumes, poultry, fish, low-fat dairy, and oils) and “unhealthy” components (red meat, processed meat, refined grains and baked products, sugar-sweetened beverages, sweets and ice cream, and fried foods). Next, we examined the frequency per day of intakes of individual foods and food groups by converting response frequencies to continuous variables using the midpoint of each response category to indicate times per day.

Mental health was assessed using the 10-item Perceived Stress Scale (PSS) and the Patient Health Questionnaire (PHQ)-4. The PSS is a widely used, validated instrument to assess individual stress levels over the last month (29). Ten questions ask respondents about how often they experienced various feelings and thoughts (e.g., been upset because of something that happened unexpectedly, felt unable to control the important things in your life). Response options range from never (0) to very often (4). A summary score is created from 0 to 40 and a score of ≥14 indicated moderate/high perceived stress. The PHQ-4 is a widely used and clinically validated four-item screener on the frequency of symptoms of anxiety and depression over the last 2 weeks (30). For anxiety, respondents indicate how often they have been bothered by feeling nervous, anxious, or on edge, and not being able to stop or control worrying. For depressive symptoms, respondents indicate how often they have been bothered by little interest or pleasure in doing things, and feeling down, depressed, or hopeless. Response options range from not at all (0) to nearly every day (3). A summary score is created from 0 to 12, and a score of ≥6 indicated moderate/severe anxiety or depression.

Finally, SNAP participants were given an opportunity to share their thoughts about the TFP benefit change in the endline survey via three open-ended questions. Specifically, they were asked: (1) How has the recent change in SNAP benefits affected your food shopping and eating behaviors?; (2) How has the recent change in SNAP benefits affected your ability to pay for other household expenses?; and (3) Is there anything else you would like to share about your experience with SNAP or food stamps since the recent change in SNAP benefits that started in October 2021? These questions were optional for current SNAP participants.

### Covariates

Covariates of interest included participants’ self-reported age (18–29, 30–39, 40–49, 50–59, ≥60 years), gender (male, female, transgender/non-binary/other), race and ethnicity (non-Hispanic White, non-Hispanic Black, non-Hispanic Asian, Hispanic, Native American/Alaskan Native/Pacific Islander/ Middle Eastern and North African/ other race/ethnicity), educational attainment (high school diploma or fewer, some college or Associate’s degree, Bachelor’s degree or higher), household income (<$25,000, $25,000- < $45,000, ≥$45,000), marital status (married or living with partner, not married or partnered), employment status (employed full-time, employed part-time, retired or unemployed), and presence of children <18 years in the home (yes, no). All covariates were assessed in the baseline (September 2021) survey.

### Analyses

In the analytic population of 1,004 adults, characteristics of SNAP participants and non-participants were first compared using likelihood ratio chi-squared tests. Within-group changes in food security status and mental health outcomes prior to and after the TFP re-evaluation were evaluated using McNemar’s test for paired nominal data. Within-group changes in dietary outcomes prior to and after the TFP re-evaluation were evaluated using paired t-tests. To assess the impact of the TFP benefit increase on SNAP participants’ outcomes, we used a difference-in-differences (DD) approach. We fit generalized linear models for the outcomes of interest with an indicator for SNAP participation, an indicator denoting the survey time (baseline vs. endline), and an interaction term between SNAP participation and survey time. Models were additionally adjusted for all sociodemographic covariates. From the DD models, we report the *P_SNAP_*, representing the significance of differences in the outcome between SNAP participants and non-participants at baseline (prior to the benefit change), *P_time_*, representing the significance of changes in the outcome from the baseline to endline surveys among non-participants, and *P_DD_*, representing the significance of the “extra” change in the outcome from the baseline to endline surveys among SNAP participants relative to concurrent changes in non-participants (i.e., the effect of the TFP re-evaluation). Statistical tests were two-sided and statistical significance was considered at *p* < 0.05. Analyzes were performed using SAS version 9.4 (SAS Institute Inc., Cary, NC).

Finally, we analyzed qualitative data from open-ended responses to the three questions about the TFP re-evaluation. Because many participants provided similar responses across the three questions, we analyzed the responses collectively rather than question-by-question. Across the three questions, we conducted inductive thematic analysis utilizing line-by-line iterative coding. Both authors reached consensus on a final set of themes and identified exemplary quotes for each theme.

## Results

### Demographic characteristics

Among the 1,004 respondents, 41% participated in SNAP at both time points and 59% did not participate in SNAP at either time points. Baseline characteristics of the respondents by SNAP participation group are shown in Table 1. There were several sociodemographic differences between these two groups: SNAP participants were more likely to be middle-aged (30–59 years), identify as female, have fewer years of education, have lower household incomes, be retired or unemployed, and have children under age 18 in the household (*P*s < 0.01) when compared to non-participants.

**Table 1:** Baseline characteristics of study participants.

	SNAP participants (*n* = 415)		Non-participants (*n* = 589)		*P*
	*n*	%	*n*	%	
Age					0.0003
18–29	55	13.3	105	17.8	
30–39	143	34.5	170	28.9	
40–49	107	25.8	120	20.4	
50–59	66	15.9	82	13.9	
60 and older	44	10.6	112	19	
Gender					<0.0001
Male	123	29.6	284	48.2	
Female	284	68.4	299	50.8	
Transgender, non-binary, or other	8	1.9	6	1	
Race/ethnicity					0.31
Non-hispanic white	293	70.6	429	72.8	
Non-hispanic Black	49	11.8	52	8.8	
Non-hispanic Asian	39	9.4	53	9.0	
Hispanic	17	4.1	36	6.1	
Native American, Pacific Islander, MENA, Other	17	4.1	19	3.2	
Education					<0.0001
High school diploma/equivalent or fewer years	86	20.7	85	14.4	
Some college or Associate’s degree	208	50.1	219	37.2	
Bachelor’s degree or higher	121	29.2	285	48.4	
Household income					<0.0001
<$25,000	241	58.1	121	20.5	
$25,000 to <$45,000	121	29.2	190	32.3	
$45,000 to <$65,000	53	12.8	278	47.2	
Marital status					0.07
Married or living with partner	161	38.8	231	39.2	
Not married or partnered	251	60.5	358	60.8	
Employment status					<0.0001
Employed full-time	111	28.5	312	54.3	
Employed part-time	124	31.8	119	20.7	
Retired or not employed	155	39.7	144	25.0	
Presence of children (<18 y) in the home					<0.0001
No	242	58.3	463	78.6	
Yes	173	41.7	126	21.4	

MENA, Middle Eastern or North African.

### Changes in food security, dietary intake, and mental health

**Figure 1:**
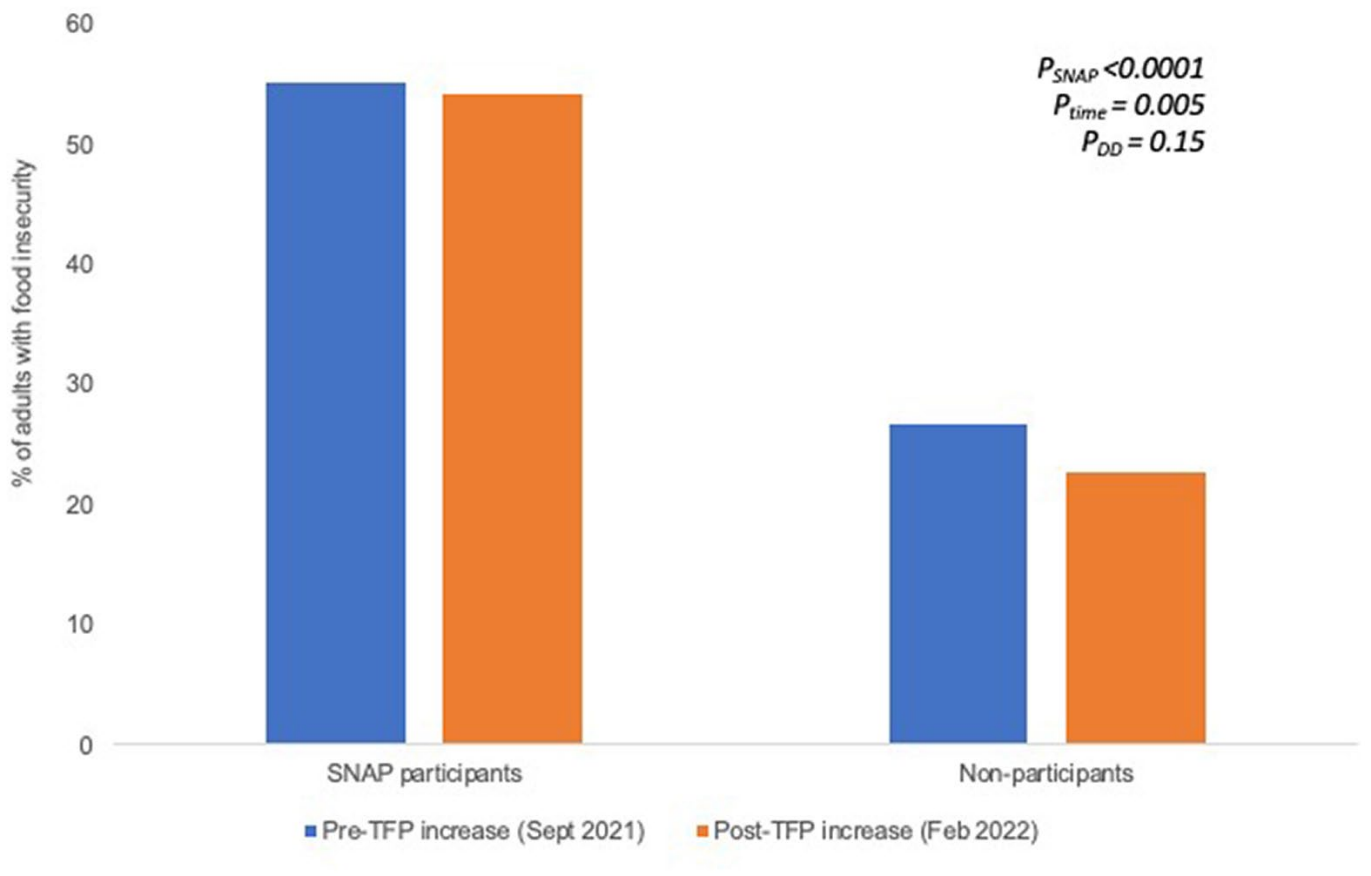
Proportion of adults with food insecurity prior to the TFP benefit increase and several months after the TFP benefit increase among SNAP participants and non-participants. Significance testing accounts for sociodemographic differences between SNAP participation groups.

Figure 1 shows changes in food security status among respondents prior to and after the TFP re-evaluation. Among SNAP participants, 55.4% experienced food insecurity at baseline (i.e., prior to the benefit change) and 55.1% experienced food insecurity at endline (i.e., after the benefit change) (*p* = 0.83). Among non-participants, 26.8% experienced food insecurity at baseline and 22.6% experienced food insecurity at endline (*p* = 0.007). Using the DD approach and adjusting for sociodemographic differences, SNAP participants had significantly higher levels of food insecurity compared to non-participants at baseline (*P*_SNAP_ < 0.0001) and non-participants had significantly improved food security from baseline to endline (*P*_time_ = 0.005). However, the TFP re-evaluation did not have a measurable impact on food insecurity among SNAP participants (*P_DD_ = 0.15*). Results were unchanged when examining the continuous HFSSM score as the outcome. Furthermore, results were unchanged when restricting the analytic population to adults with children, adults <60 years, and adults with household incomes <$45,000 (Supplementary Figures S1–S3).

Among SNAP participants, the mean PDQS scores were 52.71 (SD 11.71) prior to the benefit change and 52.50 (SD 11.21) after the benefit change (*p* = 0.58; Table 2). For non-participants, the mean PDQS scores were 54.50 (SD 11.93) at baseline and 54.95 (SD 11.42) at endline (*p* = 0.17). The DD estimates showed that SNAP participants had significantly lower overall diet quality compared to non-participants at baseline (*P*_SNAP_ = 0.05). These differences were driven by significantly lower mean unhealthy PDQS scores (reflecting higher intake) (*P*_SNAP_ = 0.0004), and in particular, higher intakes of red or processed meats (*P*_SNAP_ = 0.02) and sugar-sweetened beverages (*P*_SNAP_ < 0.0001). There were no changes in diet quality among non-participants over time (*P*_time_ = 0.31) and no differential effect of the TFP re-evaluation on diet quality by SNAP participation groups (*P*_DD_ = 0.29).

**Table 2:** Changes in dietary intake before and after the Thrifty Food Plan re-evaluation benefit change by SNAP participation groups.

	SNAP participants					Non-participants					Difference-in-difference models		
	Sept 2021 (Pre-TFP increase)		Feb 2022 (Post-TFP increase)		*P*	Sept 2021 (Pre-TFP increase)		Feb 2022 (Post-TFP increase)		*P*			
	Mean	SD	Mean	SD		Mean	SD	Mean	SD		*P_SNAP_*	*P_time_*	*P_DD_*
Overall PDQS score	52.71	11.71	52.50	11.21	0.58	54.50	11.93	54.95	11.42	0.17	0.05	0.31	0.29
PDQS score (healthy only)	25.42	10.09	24.98	9.62	0.22	25.74	10.18	25.98	10.12	0.44	0.98	0.9	0.31
PDQS score (unhealthy only)	27.3	6.78	27.52	6.62	0.34	28.77	5.83	28.97	5.69	0.28	0.0004	0.12	0.91
Dietary components (times/day)													
Vegetables	1.13	1.14	1.01	0.91	0.004	1.21	1.12	1.18	1.00	0.39	0.74	0.44	0.61
Fruits (not including juices)	0.67	0.80	0.65	0.73	0.62	0.71	0.77	0.71	0.73	0.95	0.71	0.93	0.84
Beans, peas, nuts, seeds	0.63	0.72	0.62	0.72	0.54	0.61	0.61	0.63	0.68	0.41	0.25	0.82	0.75
Poultry, fish	0.12	0.19	0.11	0.19	0.78	0.11	0.15	0.12	0.17	0.10	0.39	0.43	0.94
Red or processed meats	0.58	0.57	0.57	0.54	0.76	0.48	0.44	0.49	0.44	0.53	0.02	0.99	0.85
Whole grains	0.51	0.54	0.49	0.53	0.67	0.50	0.50	0.54	0.55	0.10	0.91	0.23	0.40
Refined grains, sweets, desserts	1.00	0.98	0.96	0.93	0.52	0.86	0.82	0.84	0.79	0.40	0.070	0.76	0.99
Sugar-sweetened beverages	0.61	0.86	0.59	0.85	0.53	0.36	0.66	0.30	0.56	0.03	<0.0001	0.22	0.25
Fried foods	0.16	0.32	0.14	0.25	0.24	0.14	0.20	0.13	0.19	0.22	0.43	0.09	0.76

TFP, Thrifty Food Plan; PDQS, Prime Diet Quality Score.

^a^Models further adjusted for age, gender, race/ethnicity, educational attainment, household income, marital status, employment status, and presence of children in the home.

**Figure 2:**
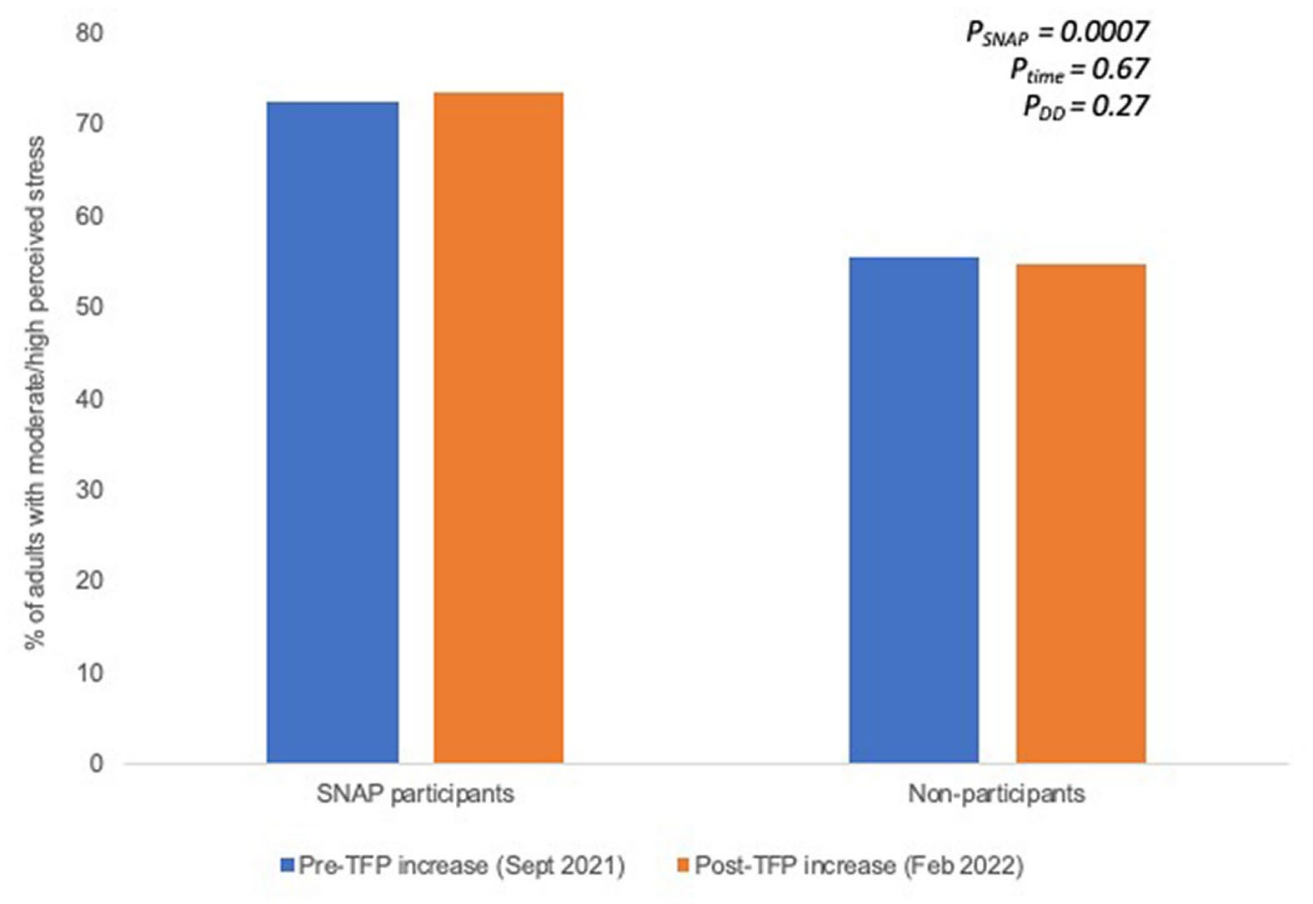
Proportion of adults with moderate/high perceived stress prior to the TFP benefit increase and several months after the TFP benefit increase among SNAP participants and non-participants. Significance testing accounts for sociodemographic differences between SNAP participation groups.

**Figure 3:**
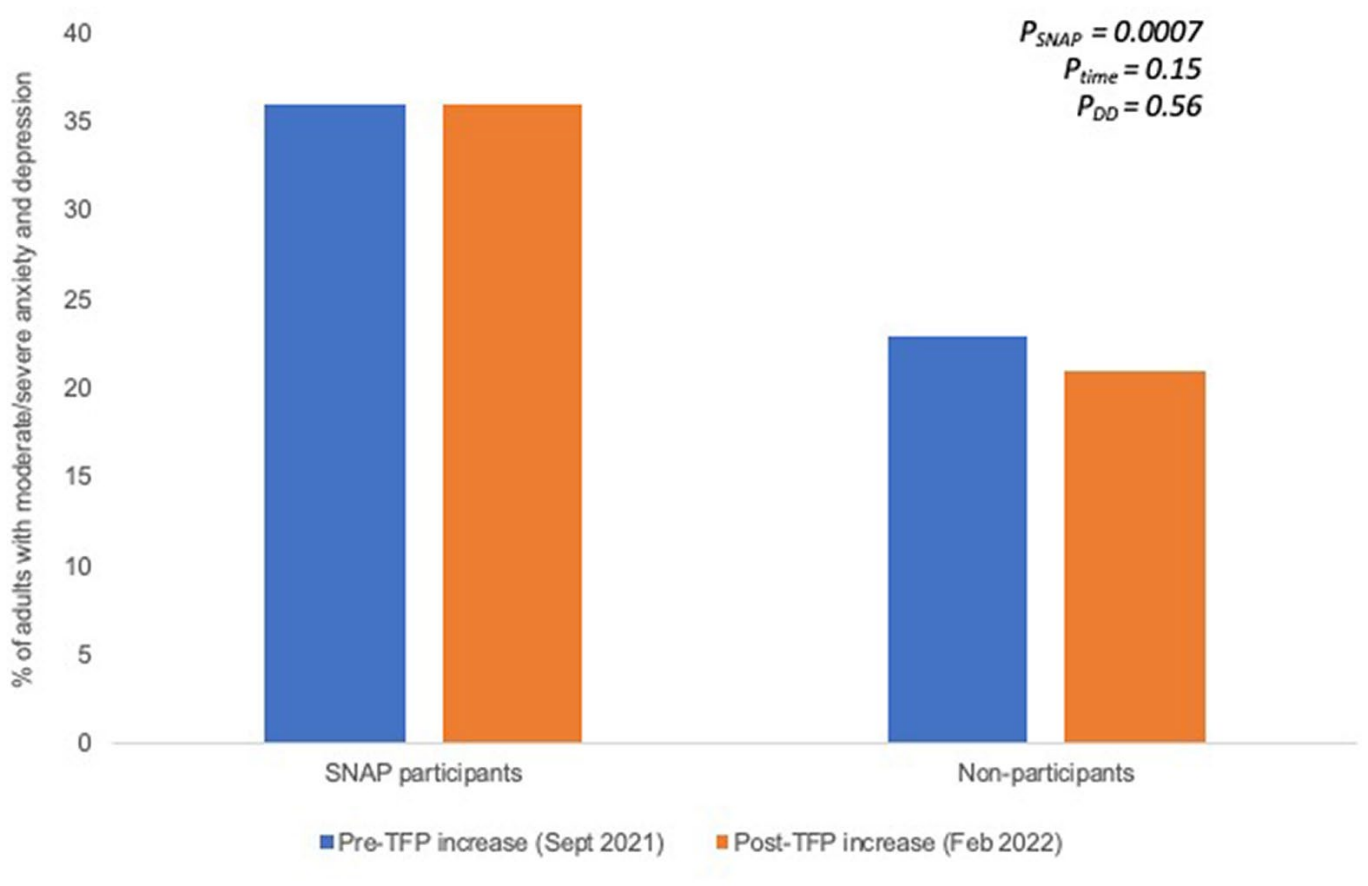
Proportion of adults with moderate/severe anxiety and depression prior to the TFP benefit increase and several months after the TFP benefit increase among SNAP participants and non-participants. Significance testing accounts for sociodemographic differences between SNAP participation groups.

Figures 2, 3 shows the changes in mental health among SNAP participants and non-participants at baseline and at endline. Among SNAP participants and non-participants, levels of moderate/high perceived stress did not significantly change over time (SNAP participants: 72.5% vs. 73.4%, *p* = 0.54; non-participants: 55.4% vs. 54.6%, *p* = 0.62; Figure 2). Similarly, among SNAP participants and non-participants, levels of moderate/severe anxiety and depression remained constant over time (SNAP participants: 36.0% vs. 36.0%, *p* = 0.91; non-participants: 22.9% vs. 21.0%, *p* = 0.16; Figure 3). Using the DD approach and adjusting for sociodemographic differences, SNAP participants had significantly higher levels of moderate/high perceived stress (*p* = 0.0007) and moderate/severe anxiety and depression (*p* = 0.0007) compared to non-participants at baseline. However, there were no significant changes in mental health outcomes among non-participants (*P*_time_ = 0.67 for perceived stress; *P*_time_ = 0.15 for anxiety/depression) and no significant impact of the TFP re-evaluation on mental health (*P*_DD_ = 0.27 for perceived stress; *P*_DD_ = 0.56 for anxiety/depression).

### Qualitative effects of the TFP re-evaluation as described by SNAP participants

Thematic analysis of the open-ended responses revealed several themes describing the impact of the TFP re-evaluation on SNAP participants’ food shopping, household spending, and food security (Table 3). Many SNAP participants described the benefit increase as coinciding with rising food prices and growing inflation, which negated the potential advantages of the benefit change. Some SNAP participants described the increase in benefits in allowing their food to stretch a few extra days, but amounts were still not enough to last the entire month. Other SNAP participants described using their extra benefits to purchase healthier, higher-quality food, particularly fruits and vegetables. SNAP participants also described the higher benefits in helping offset other household expenses, from gas and utility bills to non-food household necessities (e.g., toilet paper). Finally, some SNAP participants described the positive impact of increased SNAP benefits on their psychological distress. In particular, multiple respondents described SNAP as “a life saver” and expressed gratitude and appreciation for the program’s recent benefit increase.

**Table 3:** Responses to open-ended questions about the effects of the Thrifty Food Plan re-evaluation benefit increase in SNAP benefits from SNAP participants.

**Theme:** **Rising food prices negated the TFP increase in SNAP benefits**
“I appreciate the increase [in SNAP benefits], small though it may be, but the cost of groceries keeps going up so it really is not making a huge difference.”
“It really had no change because the increase [in benefits] coincided with an increase in groceries.”
“It has not. As a matter of fact, because of inflation, I feel like I have LESS.”
**Theme: The TFP increase helped SNAP benefits go a bit farther, but not enough to change behavior**
“I’m just able to add another day or two of meals for the month. The increase [in SNAP benefits] was only $45.”
“I do not have to skip so many days to eat something. I have some food left over for the next day(s).”
“Everything’s still the same. The raise only meant my food lasts 2.5 weeks instead of only 2 weeks.”
“I am able to buy a little more than I was prior to the change. Food last about a week more than it normally would but I still have to budget and pay cash for food.”
**Theme: The increase in SNAP benefits allow for the purchase of higher quality and/or quantity of foods**
“It has allowed me to shop for a wider variety of foods. I now try to incorporate more fruits and vegetables into my diet. I feel I can afford to eat healthier now.”
“I have been able to buy food almost every week now, whereas before the food stamps would run out by my second week. I have also been able to get more healthy food that costs more.”
“For the most part, [the increase in SNAP] has enabled me to purchase fresh and frozen fruits and vegetables more frequently.”
“It means that I can afford to purchase more organic, non-GMO foods that are healthy.”
**Theme: The increase in SNAP benefits helped to offset other household bills**
“I am able to have sufficient money to pay for things I need like toilet paper and Kleenex and paper towels because I’m not using that money for food.”
“It has allowed me to stress less about paying necessary bills like lights and internet. I do not feel like I have to work an unhealthy amount of hours just to afford to eat a decent diet.”
“[I do not have] to spend as much on food when food stamps run out so I can use that money for other things like gas.”
“I can focus on rent/cat food/electric bill and I just leave all of the food bill to however much I get in SNAP.”
**Theme: Larger SNAP benefits helped to reduce psychological stress**
“I’m just real happy to have the increase [in SNAP benefits] so I can buy better, healthier, more organic food.”
“I was able to get healthier foods, feels very uplifting.”
“I am so grateful that I’m able to get [SNAP benefits]. The increase took a good bit of stress off me.”
“[The increase in SNAP benefits] has helped me be a little more relaxed while shopping.”
“The extra money really helps families like mine, it came at a really hard time, so it was great to get that little extra money for food.”

## Discussion

In 2021, for the first time in 15 years, the TFP was updated to reflect current food prices, national dietary guidance, nutritional content, and the typical dietary patterns of low-income families (17). The TFP re-evaluation and the subsequent increased SNAP benefit levels were considered long overdue, particularly in light of the ongoing COVID-19 pandemic, rapidly changing economic conditions, rising food prices, and widening diet-related disparities. In the present study, we use the TFP re-evaluation as a natural experiment to estimate the effect of this policy on SNAP participants’ health and well-being. Given the recency of the TFP update, to our knowledge, this is the first study to examine the impact of this policy change on adult SNAP participants’ food insecurity, diet quality, and mental health outcomes.

Our results highlight two main findings. First, SNAP participants are demonstrably worse off than non-participants with respect to every measurable outcome–higher food insecurity, lower diet quality, greater perceived stress, and higher anxiety and depressive symptoms, even after accounting for observable sociodemographic differences between the two groups. This indicates that even in our income-limited study population, those who choose to participate in SNAP are prone to greater food and economic hardships, and are most vulnerable to food insecurity and its adverse health outcomes. While these differences have been previously documented, results of the current study show that these disparities continue to persist, even with the unprecedented expansion of SNAP during the COVID-19 pandemic (31–34).

Second, the increase in benefits from the TFP re-evaluation yielded no significant changes in SNAP participants’ food security, dietary intake, or mental health outcomes compared to non-participants over the same time period. One interpretation of this finding may be that the TFP re-evaluation had no impact. However, thematic analysis from the open-ended responses show that SNAP participants described modest improvements in their ability to purchase higher quality and/or quantity of food. Furthermore, participants reported that higher SNAP benefits helped to offset higher costs for other basic needs (i.e., housing and energy bills), which could have prevented additional stress and economic instability. However, given rapidly rising food prices, the overall increase was insufficient to influence their dietary habits or mental health. A more plausible interpretation of the study’s findings is that the TFP benefit increase, while not high enough to improve SNAP participants’ outcomes compared to non-participants, did help to prevent inflation-related disparities in food insecurity and health outcomes from widening among SNAP participants.

In this vein, it important to recognize that the SNAP benefit boost from the TFP re-evaluation occurred during the middle of the COVID-19 pandemic when SNAP and other food assistance programs underwent significant expansions to their benefit levels, program structure, and eligibility and recertification processes (35, 36). At the same time, there were historic changes to economic and health programs like the expansion of the Earned Income Tax Credit, the extension of unemployment benefits, the expansion of health insurance programs, and the provision of economic impact (i.e., stimulus) payments, all of which contributed to alleviating food and economic hardship in lower-income households (37–39). The complicated economic, policy, and health layers of the COVID-19 landscape pose unique challenges to examining the effects of the TFP benefit increase compared to had the policy change occurred in a non-pandemic setting.

SNAP benefits have long been recognized as insufficient to ensure adequate and nutritious food throughout the month. An in-depth report conducted by the USDA showed that unaffordability of healthy food was the most significant barrier to SNAP benefit adequacy (40). Compared to the 2006 TFP, the 2021 update led to a 21% increase in SNAP benefits. Although seemingly substantial, the change in benefits was only approximately $36.30 per month for a family of four, or $1.20 per person a day (17). The Urban Institute projected that the TFP-related SNAP benefit increase would reduce poverty by 4.7% and child poverty by 8.6% (41). However, the benefit change from the TFP re-evaluation was implemented during a period with inflation and food prices at historic highs. The U.S. Bureau of Labor Statistics’ Consumer Price Index, an economy-wide inflation measure, showed that total food prices grew 6.1% from November 2020 to November 2021 and 10.6% from November 2021 to November 2022, with staple items like meats, fruit, and eggs being most affected (42, 43). Results of the current study align with anecdotal evidence of the impact of high food prices on food insecurity among SNAP participants (44). In order to detect significant improvements in SNAP participants’ outcomes relative to non-participants, greater increases to SNAP benefits may be needed to capture the seasonal and geographic variability of food prices. Additionally though SNAP benefits are annually adjusted for food-related inflation, during periods of economy-wide high inflation such as the period from late 2021–2022, more frequent or robust adjustments to SNAP benefit levels may be warranted (45).

Our findings have important policy implications. The upcoming Farm Bill reauthorization provides a critical opportunity to further strengthen SNAP. The structure of SNAP as an entitlement program allows it to respond to major economic shocks swiftly and effectively. During the COVID-19 pandemic, the unprecedented expansion of SNAP helped low-income Americans to access and afford food during widespread economic instability, showing the political support and feasibility of these legislative actions (8, 46). As several of these policies, including the extra benefits provided via emergency allotments, have ended by May 2023, there is considerable and well-founded concern that a substantial loss of SNAP benefits will lead to more prevalent and severe levels of food insecurity, and the physical and mental consequences that stem from it (47–49). A report from the Census Bureau estimates that the expiration of emergency allotments will result in a $600/month reduction in SNAP benefits for a four-person household with a net monthly income of $2,000 (50). Food insecurity persists as a national health challenge and SNAP continues to be the cornerstone program to combat food insecurity and poverty in the U.S. In addition to the permanent increase in SNAP benefits from the TFP re-evaluation, other actions that the U.S. Congress can take to strengthen SNAP in the next Farm Bill include: (1) incentivize the purchase of healthier foods (e.g., fruits and vegetables), (2) allow hot and prepared foods with program benefits, (3) subsidize online grocery delivery fees for SNAP participants, (4) provide greater nutrition education, and (5) suspend work requirements for certain demographic groups to facilitate SNAP participation (e.g., college students). All of these strategies have garnered broad bipartisan support from low-income Americans (51). Furthermore, the U.S. Department of Agriculture should explore incentives for these retailers accepting SNAP benefits to stock and sell nutritious foods at affordable prices or re-implement the stricter stocking standards for retailers authorized to receive SNAP benefits as was previously established in 2016 and later tabled (52). Such policies would result in improved nutritious food access for both SNAP participants and non-participants in low-income communities without easy access to large grocery stores, as parallel studies from the WIC food package revision have shown (53, 54).

Strengths of our study include the large sample of survey respondents, timely data collection spanning the month before to 4 months after the SNAP benefit increase, use of validated instruments for outcome assessment, and inclusion of qualitative responses to supplement quantitative findings. Our study also has several limitations. First, our study population is a convenience sample recruited through CloudResearch. Thus, our findings may not be generalizable to the larger populations of low-income adults or SNAP participants. Both surveys were web-based, which limited participation to those with a computer or mobile device, with an Internet connection, and with the technological expertise to use MTurk. The surveys were also conducted in English, which may have excluded minority racial and ethnic adults with limited English proficiency. Another limitation is that we relied on self-reported SNAP participation to define the exposure. This may have resulted in some SNAP participants being misclassified; however, this was the most feasible approach given the varying state-specific SNAP eligibility criteria as a result of the ongoing COVID-19 pandemic. Similarly, our study did not assess all the factors that contribute to SNAP eligibility (e.g., assets, household members with disabilities) and non-participants may include individuals in income-eligible and income-ineligible households. Furthermore, due to several pandemic-related national and state-specific changes to SNAP, benefit allotments may have increased or decreased over the same time period for reasons other than the TFP re-evaluation. Finally, the follow-up surveys were collected 4 months after the benefit increase from the TFP re-evaluation went into effect. A longer follow-up period may be needed to see significant policy effects. However, the majority of SNAP participants do not consistently receive benefits over a long period of time, and those who do consistently receive benefits could be systematically different compared to those who stop participating, which could complicate longer-term follow-up studies. Additional research with large and nationally representative samples are needed to understand the broad effects of this policy change on SNAP participants’ health and well-being.

## Conclusion

The long overdue modernization of the TFP by the USDA in 2021 resulted in a critical and permanent increase in SNAP benefits amidst the COVID-19 pandemic. However, results from the current study show no significant effects of the TFP-related benefit increase on food insecurity, diet quality, or mental health of adult SNAP participants, compared to low-income, non-participants, suggesting that the increased benefits were still insufficient to improve food security and health behaviors in this convenience sample. High inflation during the study period is important to contextualize these results; historically high food prices may have undermined the short-term impact of the TFP-related increase to SNAP benefits. Further research is needed to determine the national and long-term impacts of this policy change, in combination with other pandemic-related SNAP expansion policies, on the health and well-being of key demographic groups participating in SNAP.

## Data availability statement

The raw data supporting the conclusions of this article will be made available by the authors by request.

## Ethics statement

The studies involving human participants were reviewed and approved by University of Michigan IRB Health Sciences and Behavioral Sciences. The participants provided their written informed consent to participate in this study.

## Author contributions

CL and JW conceptualized and designed the study and collected the data. CL performed statistical analysis and wrote the first draft of the manuscript. JW contributed to manuscript revision. All authors have read and approved the submitted version.

## Funding

This project was supported by a grant from the Johns Hopkins University Bloomberg American Health Initiative Mid-year Funding Program and faculty research funds from the University of Michigan. JW was also supported by the National Institutes of Diabetes and Digestive and Kidney Diseases (K01DK119166).

## Conflict of interest

The authors declare that the research was conducted in the absence of any commercial or financial relationships that could be construed as a potential conflict of interest.

## Publisher’s note

All claims expressed in this article are solely those of the authors and do not necessarily represent those of their affiliated organizations, or those of the publisher, the editors and the reviewers. Any product that may be evaluated in this article, or claim that may be made by its manufacturer, is not guaranteed or endorsed by the publisher.

## Supplementary material

The Supplementary material for this article can be found online at: https://www.frontiersin.org/articles/10.3389/fpubh.2023.1142577/full#supplementary-material

## References

[1] Wolfson JA Leung CW. Food insecurity and COVID-19: disparities in early effects for US adults. Nutrients. (2020) 12:1648. doi: 10.3390/nu12061648, PMID: 32498323 PMC7352694

[2] Fitzpatrick KM Harris C Drawve G Willis DE. Assessing food insecurity among US adults during the COVID-19 pandemic. J Hunger Environ Nutr. (2021) 16:1–18. doi: 10.1080/19320248.2020.1830221

[3] Niles MT Bertmann F Belarmino EH Wentworth T Biehl E Neff R. The early food insecurity impacts of COVID-19. Nutrients. (2020) 12:2096. doi: 10.3390/nu12072096, PMID: 32679788 PMC7400862

[4] Lauren BN Silver ER Faye AS Rogers AM Woo-Baidal JA Ozanne EM. Predictors of households at risk for food insecurity in the United States during the COVID-19 pandemic. Public Health Nutr. (2021) 24:3929–36. doi: 10.1017/S1368980021000355, PMID: 33500018 PMC8207551

[5] Coleman-Jensen A Rabbitt MP Gregory CA Singh A. Household food security in the United States in 2021, ERR-309. Economic Research Service. Washington, DC: US Department of Agriculture (2022).

[6] Laraia B. Food insecurity and chronic disease. Adv Nutr. (2013) 4:203–12. doi: 10.3945/an.112.003277, PMID: 23493536 PMC3649100

[7] Bruening M Dinour LM Chavez JBR. Food insecurity and emotional health in the USA: a systematic narrative review of longitudinal research. Public Health Nutr. (2017) 20:3200–8. doi: 10.1017/S1368980017002221, PMID: 28903785 PMC10261670

[8] Toossi S Jones JW Hodges L. The food and nutrition assistance landscape: fiscal year 2020 annual report (EIB-227). Economic Research Service. Washington, DC: US Department of Agriculture. (2021).

[9] Institute of Medicine and National Research Council. Supplemental nutrition assistance program: Examining the evidence to define benefit adequacy. Washington, DC: The National Academies Press (2013). 24901188

[10] Nord M. How much does the supplemental nutrition assistance program alleviate food insecurity? Evidence from recent programme leavers. Public Health Nutr. (2012) 15:811–7. doi: 10.1017/S1368980011002709, PMID: 22015063

[11] Bailey MJ Hoynes HW Rossin-Slater M Walker R. Is the social safety net a long-term investment? Large-scale evidence from the food stamp program (NBER working paper no. 26942). Cambridge, MA: National Bureau of Economic Research (2020). 10.1093/restud/rdad063 PMC11395512 39281422

[12] Canning P Stacy B. The supplemental nutrition assistance program (SNAP) and the economy: new estimates of the SNAP multiplier, ERR-265. Economic Research Service. Washington, DC: US Department of Agriculture. (2019).

[13] H.R.6201–116th Congress. *Families first coronavirus response act (2019-2020)*. (2020). Available at: https://www.congress.gov/bill/116th-congress/house-bill/6201/text. (Accessed March 18, 2020).

[14] H.R.1319–117th Congress. *American rescue plan act of 2021 (2021-2022)*. (2021) Available at: https://www.congress.gov/bill/117th-congress/house-bill/1319/text. (Accessed March 11, 2021).

[15] Economic Relief Related to the COVID-19 Pandemic. *Economic Relief Related to the COVID-19 Pandemic (Executive Order 14002) (2021–01923)*. (2021).

[16] SNAP and the Thrifty Food Plan. *Food and nutrition services*, US Department of Agriculture (2022). Available at: https://www.fns.usda.gov/snap/thriftyfoodplan.

[17] Thrifty Food Plan, 2021 (FNS-916). Food and Nutrition Service. Washington, DC: US Department of Agriculture (2021).

[18] USDA. Modernizes the Thrifty Food Plan, Updates SNAP Benefits. First update in more than 45 years reflects current cost realities: Food and Nutrition Service. Washington, DC: US Department of Agriculture (2021).

[19] Litman L Robinson J Abberbock T. TurkPrime.com: a versatile crowdsourcing data acquisition platform for the behavioral sciences. Behav Res Methods. (2017) 49:433–42. doi: 10.3758/s13428-016-0727-z, PMID: 27071389 PMC5405057

[20] Smith NA Sabat IE Martinez LR Weaver K Xu S. A convenient solution: using MTurk to sample from hard-to-reach populations. Ind Org Psychol. (2015) 8:220–8. doi: 10.1017/iop.2015.29

[21] Huff C Tingley D. “Who are these people?” evaluating the demographic characteristics and political preferences of MTurk survey respondents. Res Pol. (2015) 2:560464. doi: 10.1177/2053168015604648

[22] Wolfson JA Lahne J Raj M Insolera N Lavelle F Dean M. Food Agency in the United States: associations with cooking behavior and dietary intake. Nutrients. (2020) 12. doi: 10.3390/nu12030877, PMID: 32213985 PMC7146410

[23] Leung CW Wolfson JA. Perspectives from supplemental nutrition assistance program participants on improving SNAP policy. Health Equity. (2019) 3:81–5. doi: 10.1089/heq.2018.0094, PMID: 30915423 PMC6434592

[24] Russell AM Barry AE. Psychometric properties of the AUDIT-C within an Amazon mechanical Turk sample. Am J Health Behav. (2021) 45:695–700. doi: 10.5993/AJHB.45.4.8, PMID: 34340736

[25] Zimmerman M Kerr S. How should the severity of depression be rated on self-report depression scales? Psychiatry Res. (2019) 280:112512. doi: 10.1016/j.psychres.2019.112512 31425849

[26] Bowen HJ Gallant SN Moon DH. Influence of reward motivation on directed forgetting in younger and older adults. Front Psychol. (2020) 11:1764. doi: 10.3389/fpsyg.2020.01764, PMID: 32849044 PMC7411084

[27] U.S. Household Food Security Survey Module: Three-Stage Design, With Screeners. Economic Research Service. Washington, DC: US Department of Agriculture (2012).

[28] Bromage S Batis C Bhupathiraju SN Fawzi WW Fung TT Li Y. Development and validation of a novel food-based global diet quality score (GDQS). J Nutr. (2021) 151:75S–92S. doi: 10.1093/jn/nxab244, PMID: 34689200 PMC8542096

[29] Cohen S. Perceived stress in a probability sample of the United States In: Spacapan S Oskamp S, editors. The Claremont symposium on applied social psychology. The social psychology of health. Thousand Oaks, CA: Sage Publications, Inc. (1988)

[30] Kroenke K Spitzer RL Williams JB Lowe B. An ultra-brief screening scale for anxiety and depression: the PHQ-4. Psychosomatics. (2009) 50:613–21. doi: 10.1176/appi.psy.50.6.613, PMID: 19996233

[31] Leung CW Willett WC Ding EL. Low-income supplemental nutrition assistance program participation is related to adiposity and metabolic risk factors. Am J Clin Nutr. (2012) 95:17–24. doi: 10.3945/ajcn.111.012294, PMID: 22170370 PMC3238460

[32] Andreyeva T Tripp AS Schwartz MB. Dietary quality of Americans by supplemental nutrition assistance program participation status: a systematic review. Am J Prev Med. (2015) 49:594–604. doi: 10.1016/j.amepre.2015.04.035, PMID: 26238602 PMC6022372

[33] Chaparro MP Harrison GG Pebley AR Wang M. The relationship between obesity and participation in the supplemental nutrition assistance program (SNAP): is mental health a mediator? J Hunger Environ Nutr. (2014) 9:512–22. doi: 10.1080/19320248.2014.962780, PMID: 26413180 PMC4580337

[34] Siddiqi SM Cantor J Dastidar MG Beckman R Richardson AS Baird MD. SNAP participants and high levels of food insecurity in the early stages of the COVID-19 pandemic. Public Health Rep. (2021) 136:457–65. doi: 10.1177/00333549211007152, PMID: 33789530 PMC8203047

[35] Center on Budget and Policy Priorities. States are using much-needed temporary flexibility in SNAP to respond to COVID-19 challenges. Washington, DC: Center on Budget and Policy Priorities (2021).

[36] US Department of Agriculture. *SNAP Extension of COVID-19 Administrative Flexibilities: May 2021 and Beyond*. Food and Nutrition Service. US Department of Agriculture. (2021). Available at: https://www.fns.usda.gov/snap/extension-covid-19-administrative-flexibilities-may-2021-and-beyond.

[37] Shafer PR Gutierrez KM Ettinger de Cuba S Bovell-Ammon A Raifman J. Association of the Implementation of child tax credit advance payments with food insufficiency in US households. JAMA Netw Open. (2022) 5:e2143296. doi: 10.1001/jamanetworkopen.2021.43296, PMID: 35024837 PMC8759005

[38] Ganong P Noel P Vavra J. US unemployment insurance replacement rates during the pandemic. J Public Econ. (2020) 191:104273. doi: 10.1016/j.jpubeco.2020.104273, PMID: 33012869 PMC7525248

[39] Center on Budget and Policy Priorities. Robust COVID relief achieved historic gains against poverty and hardship, bolstered economy. Washington, DC: Center on Budget and Policy Priorities (2022).

[40] Gearing M Dixit-Joshi S May LB. Arriers that constrain the adequacy of supplemental nutrition assistance program (SNAP) allotments. Washington, DC: Westat Inc. for the US Department of Agriculture, Food and Nutrition Service (2021).

[41] Wheaton L Kwon D. Effect of the reevaluated thrifty food plan and emergency allotments on supplemental nutrition assistance program benefits and poverty. Washington, D.C: Urban Institute (2022).

[42] US Department of Labor. Bureau of Labor Statistics, U.S. Department of Labor. Consumer Price Index–November 2021 (USDL-21-2101). Washington, DC: US Department of Labor (2021).

[43] Consumer Price Index - November 2022 (USDL-22-2304). Bureau of Labor Statistics. Washington, DC: US Department of Labor (2022).

[44] Cronin D. *‘It’s not enough.’ SNAP Recipients Struggle Amid High Food Prices. Civil Eats*. (2022). Available at: https://civileats.com/2022/12/12/its-not-enough-snap-recipients-struggle-amid-high-food-prices/.

[45] Llobrera J. SNAP benefit adjustments will help low-income households cope with food inflation. Washington, DC: Center on Budget and Policy Priorities (2022).

[46] Improving food and nutrition security during COVID-19, the economic recovery, and beyond. Washington, DC: Bipartisan Policy Center (2021).

[47] Ettinger de Cuba S Chilton M Bovell-Ammon A Knowles M Coleman SM Black MM. Loss of SNAP is associated with food insecurity and poor health in working families with young children. Health Aff (Millwood). (2019) 38:765–73. doi: 10.1377/hlthaff.2018.05265, PMID: 31059367

[48] Ettinger de Cuba S Harker L Weiss I Scully K Chilton M Coleman S. Punishing hard work: The unintended consequences of cutting SNAP benefits. Boston, MA: Children's HealthWatch (2013).

[49] Katare B Kim J. Effects of the 2013 SNAP benefit cut on food security. Appl Econ Perspect Policy. (2017) 39:662–81. doi: 10.1093/aepp/ppx025

[50] Brown AR Giefer KG King MD. Impact of the end of extra SNAP benefits. Washington, DC: US Census Bureau (2023).

[51] Wolfson JA Leung CW Moran A. *Meeting the moment: policy changes to strengthen SNAP and improve health*. Milbank Quarterly Opinion (2021).

[52] SNAP. *Enhancing retailer standards in the supplemental nutrition assistance program (SNAP)*. FNS-2016-0018241. (2016).

[53] Rose D O'Malley K Dunaway LF Bodor JN. The influence of the WIC food package changes on the retail food environment in New Orleans. J Nutr Educ Behav. (2014) 46:S38–44. doi: 10.1016/j.jneb.2014.01.008, PMID: 24809995

[54] Hillier A McLaughlin J Cannuscio CC Chilton M Krasny S Karpyn A. The impact of WIC food package changes on access to healthful food in 2 low-income urban neighborhoods. J Nutr Educ Behav. (2012) 44:210–6. doi: 10.1016/j.jneb.2011.08.004, PMID: 22405817

