# Supplementary material for: The impact of the 2021 Thrifty Food Plan benefit re-evaluation on SNAP participants’ short-term food security and health outcomes
Source: Front Public Health. 2023 Jun 29;11:1142577. doi: 10.3389/fpubh.2023.1142577 (PMC10343438; doi:10.3389/fpubh.2023.1142577)

**Supplemental Figure 1: Proportion of adults with children with food insecurity prior to the TFP benefit increase and several months after the TFP benefit increase among SNAP participants and non-participants. Significance testing accounts for sociodemographic differences between SNAP participation groups.**


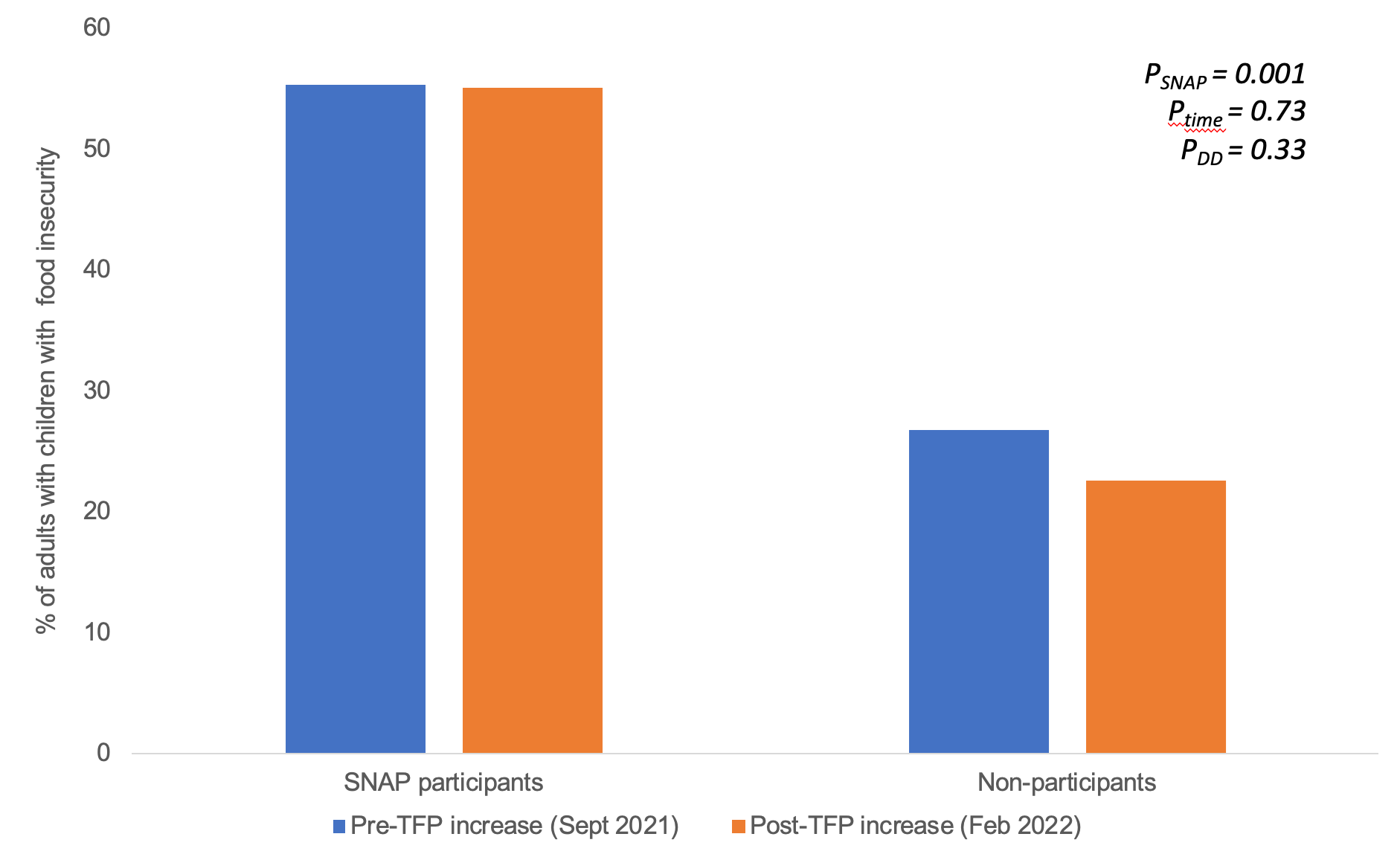


**Supplemental Figure 2: Proportion of adults <60 years with food insecurity prior to the TFP benefit increase and several months after the TFP benefit increase among SNAP participants and non-participants. Significance testing accounts for sociodemographic differences between SNAP participation groups.**


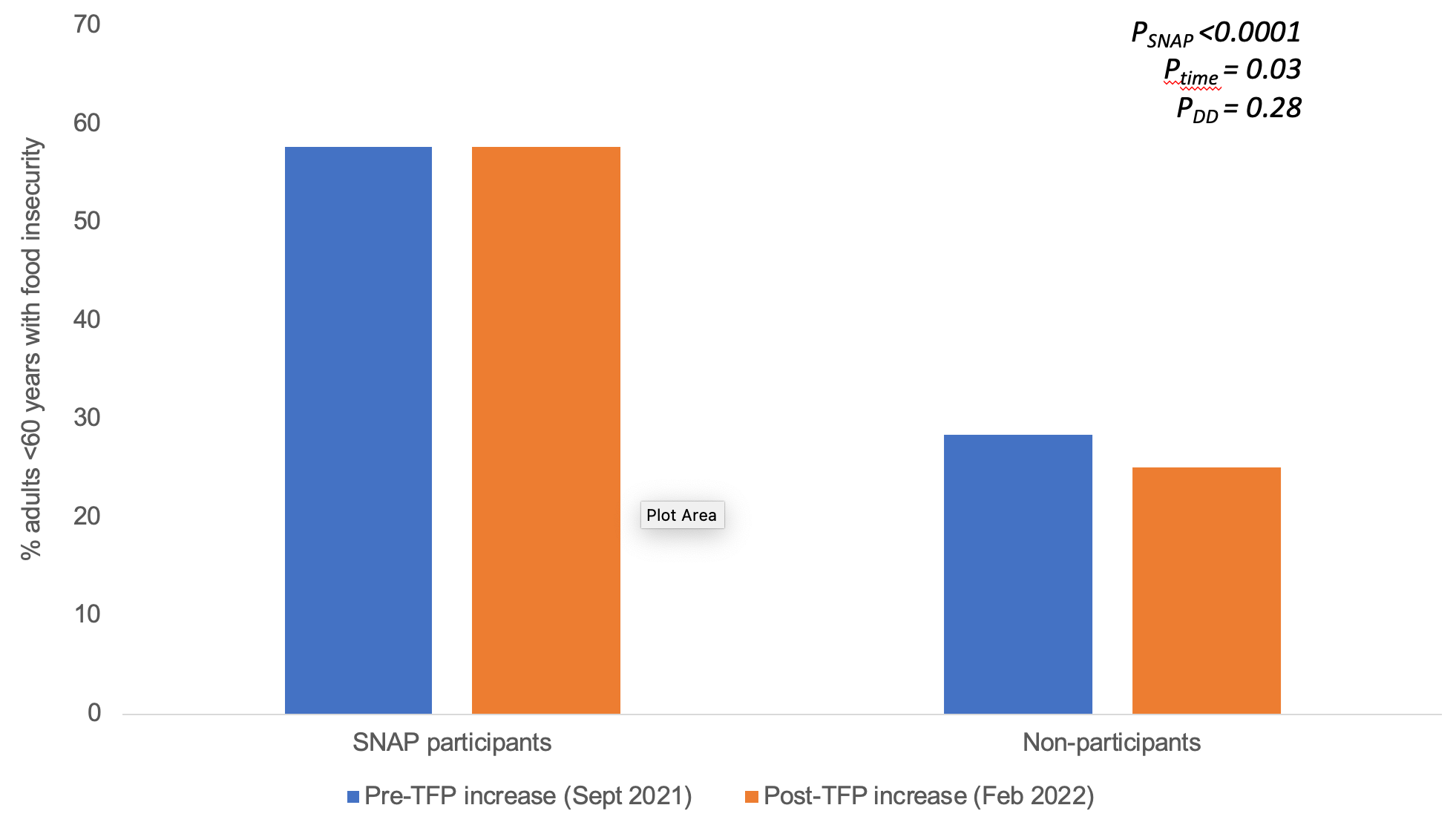


**Supplemental Figure 3: Proportion of adults with incomes <$45,000 with food insecurity prior to the TFP benefit increase and several months after the TFP benefit increase among SNAP participants and non-participants. Significance testing accounts for sociodemographic differences between SNAP participation groups.**


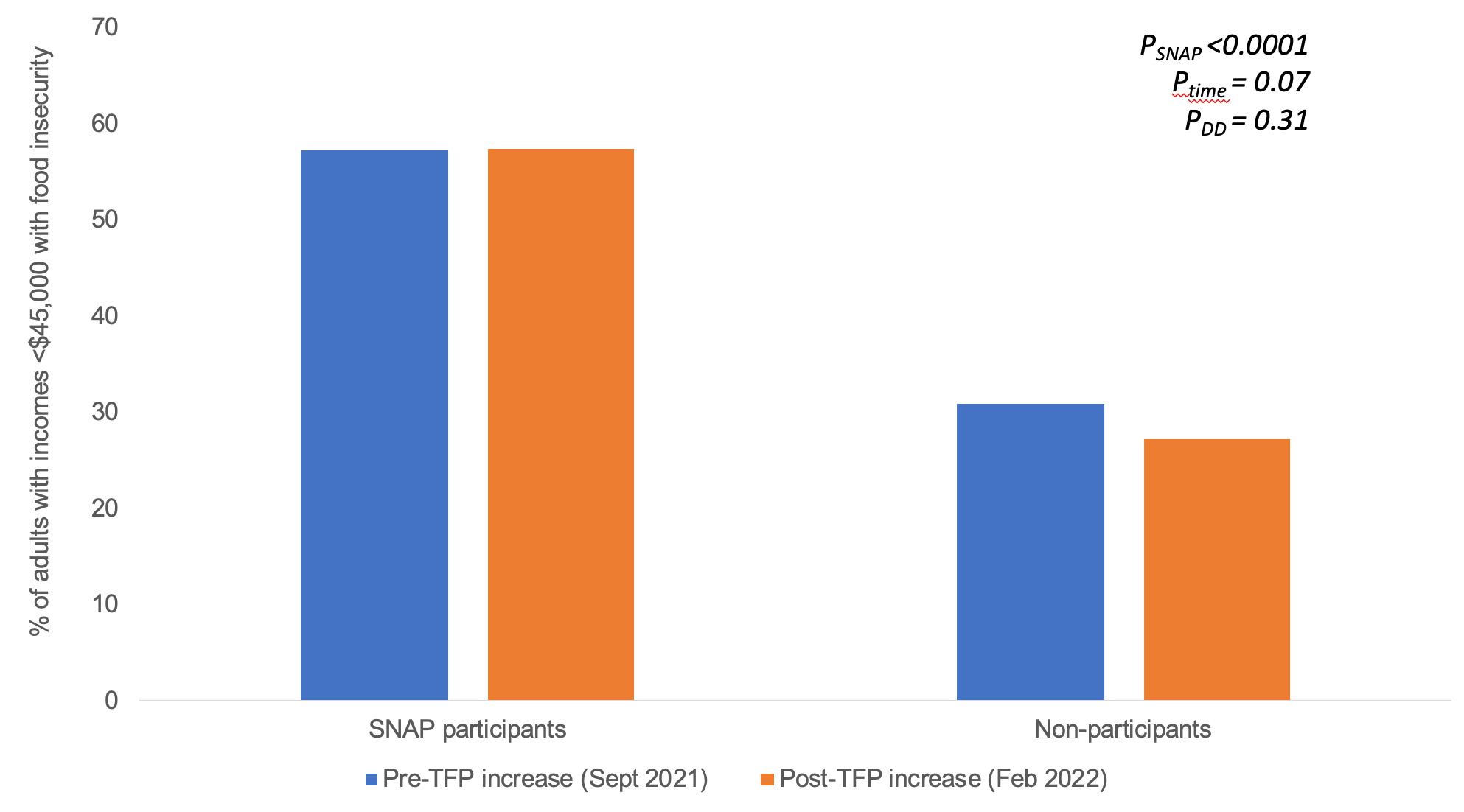

Supplement: Supplementary file 1 [file Data_Sheet_1.docx]
